# Extra-articular Soft Arthroscopic Latarjet Technique: More Versatility and Closer Reproducibility of Classic Latarjet Procedure than Its Intra-articular Counterpart

**DOI:** 10.1016/j.eats.2021.05.009

**Published:** 2021-08-02

**Authors:** Amr Abdel-Mordy Kandeel

**Affiliations:** Department of Orthopedics and Traumatology, Faculty of Medicine, Menoufia University, Shebien El-kom, Menoufia Governorate, Egypt

## Abstract

The recent innovative concept of dynamic anterior stabilization of the shoulder by long head of biceps tendon for anterior gleno-humeral instability management has gained growing popularity among shoulder surgeons. Different techniques using this concept have been reported. Nevertheless, these techniques share common steps of tenotomy, re-routing, trans-subscapularis transfer and bony glenoid fixation of long head of biceps. Lately, a simplified procedure of intra-articular soft arthroscopic Latarjet technique has been introduced to refer to soft tissue tenodesis of long head of biceps to subscapularis tendon by 2 simple stitches of nonabsorbable sutures following Bankart repair. For more technical simplicity and closer reproducibility of gleno-humeral restabilization mechanisms of Latarjet procedure; the current Technical Note describes the extra-articular soft arthroscopic Latarjet technique, whereby long head of biceps is retrieved to the subpectoral region following intra-articular tenotomy; whip-stitched; rerouted deep to pectoralis major; and passed within subscapularis window into the gleno-humeral joint, where it is sutured over itself around upper subscapularis tendon. The currently reported technique offers potential advantages of versatility, steep learning curve, low cost (no hardware), feasibility of concurrent gleno-humeral restabilization procedures, and technical easiness of revision management; however; it is nonanatomic and should be biomechanically and clinically investigated to validate its long-term versatile utility.

## Introduction

Over the past decade, a paradigm shift has evolved for management of anterior gleno-humeral (GH) instability through arthroscopic reproduction of the Latarjet procedure. Notably, in 2007, Lafosse et al. described an arthroscopic technique of trans-subscapularis coracoid graft transfer and fixation into the deficient antero-inferior glenoid.^1^, ^2^, ^3^, ^4^, ^5^, ^6^, ^7^, ^8^

On other hand, different techniques of dynamic anterior stabilization (DAS) of the shoulder using long head of biceps (LHB) tendon as an alternate sling to that of the conjoint tendon of Latarjet procedure have been introduced. However, these different techniques share common features of arthroscopic tenotomy, trans-subscapularis rerouting, and anterior glenoid fixation of LHB tendon.^9^, ^10^, ^11^

For simplification of DAS techniques, intra-articular soft arthroscopic Latarjet technique (in-SALT) was recently reported; in which Bankart repair is followed by soft tissue tenodesis of tenotomized LHB tendon to upper border of subscapularis (SSC) tendon by 2 simple stitches of nonabsorbable sutures.^12^

**Fig 1:**
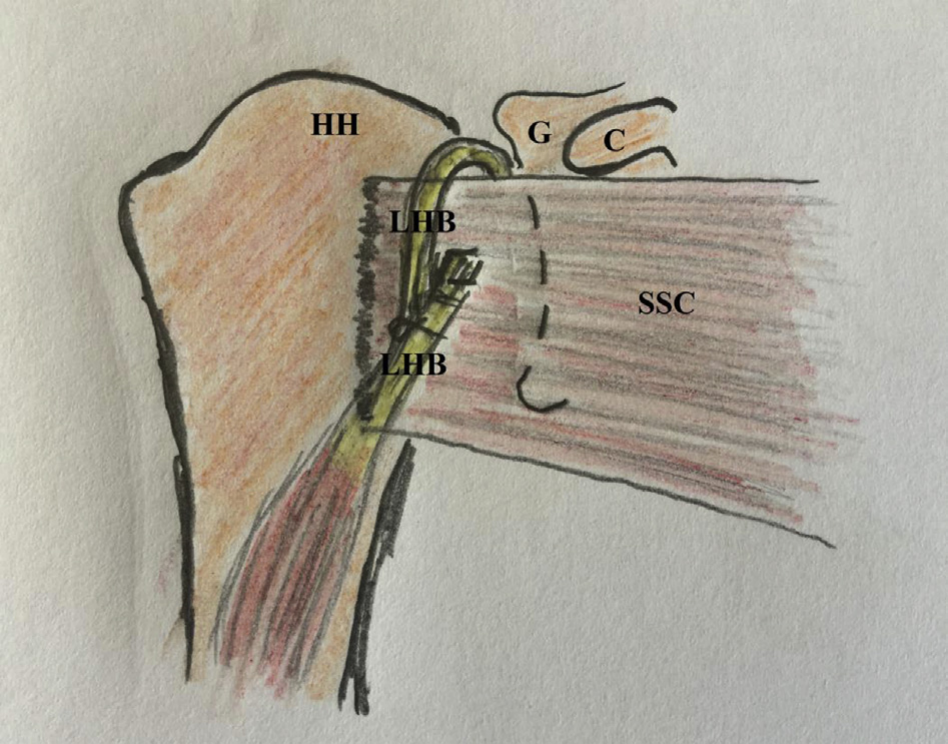
Technical principle of extra-articular soft arthroscopic Latarjet technique (ex-SALT) in the right shoulder. Long head of biceps tendon is identified in the subpectoral region, tenotomized from its superior labral origin, retrieved to the subpectoral region, whip-stitched by nonabsorbable sutures, rerouted deep to the pectoralis major muscle to SSC, passed within a SSC window, and sutured over itself around upper border of SSC. C, coracoid process; G, glenoid; HH, humeral head; LHB, long head of biceps tendon; SSC, subscapularis muscle.

For more technical convenience and closer reproducibility of Latarjet dynamic-stabilizing mechanisms, the current article describes extra-articular soft arthroscopic Latarjet technique (ex-SALT). This is an alternative GH re-stabilization technique in which LHB tendon is identified and suture-marked in the subpectoral region, tenotomized from its superior labral origin, retrieved to the subpectoral region, whip-stitched by nonabsorbable sutures, rerouted deep to the pectoralis major (PM) muscle to SSC, passed within a SSC window, and sutured over itself around upper border of SSC. Fig 1 depicts technical principle of the reported technique.

## Operative Technique

The current Technical Note describes ex-SALT for anterior GH instability management in patients with extensive anterior labral detachments (i.e., type V superior labrum from anterior to posterior [SLAP] lesion), antero-inferior GH capsulo-labral deficiency and revision procedures. The study was approved by the Institutional Committee of Scientific Research and Ethics of Faculty of Medicine, Menoufia University, Egypt.

### Setup

**Fig 2:**
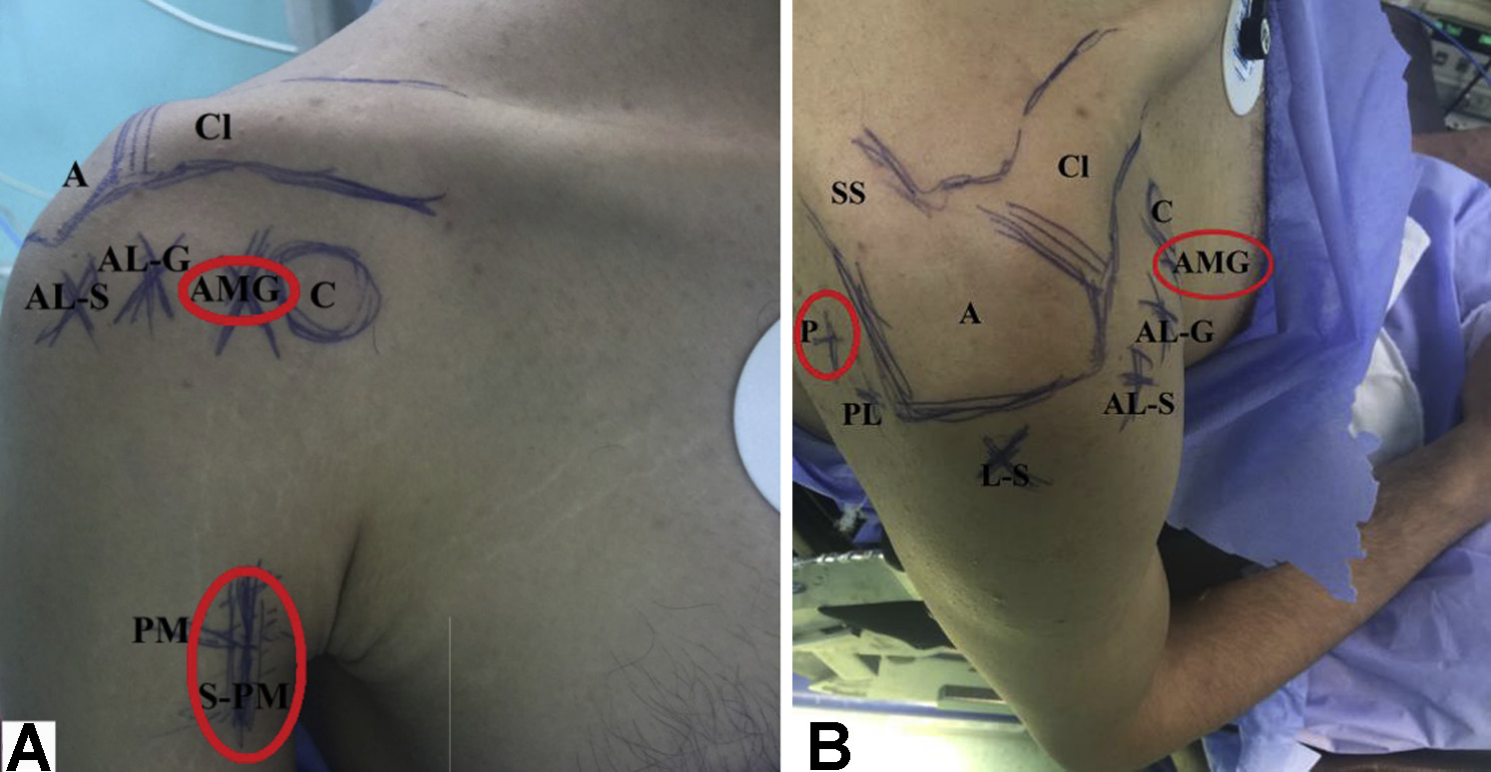
(A and B) Anatomic landmarks marked (in red ovals) for extra-articular soft arthroscopic Latarjet technique (ex-SALT) in the right shoulder. (A) Anterior shoulder aspect. (B) Lateral and posterior shoulder aspects. A, acromion; AL-G, antero-lateral gleno-humeral portal; AL-S, antero-lateral sub-acromial portal; AMG, anterior mid-glenoid portal; C, coracoid process; Cl, clavicle (lateral end); L-S, lateral sub-acromial portal; P, standard posterior gleno-humeral portal; PL, postero-lateral sub-acromial portal; PM, pectoralis major muscle (lower border); SS, scapular spine; S-PM, subpectoral approach.

Initial setup includes general anesthesia, prophylactic antibiotic administration, beach-chair positioning, and marking of bony and soft tissue anatomic landmarks. Fig 2, A and B, show marked anatomic landmarks for the reported technique.

### Examination Under Anesthesia

The operated shoulder is examined for evaluation of range of motion (ROM), determination of instability direction and grading, and assessment of engagement of Hill-Sachs lesion over the anterior glenoid by placing the shoulder in the provocative position of 90°-90° abduction-external rotation.

### Diagnostic Arthroscopic GH Examination

Arthroscopic GH examination is performed via standard viewing posterior, and then, working anterior mid-glenoid portals for diagnosis of soft tissue and bony GH lesions. Assessment of bony lesions includes quantification of anterior glenoid bone loss using a calibrated probe to rule out critical glenoid bone deficiency (>20%). In addition, engagement of Hill-Sachs lesion was arthroscopically evaluated while the shoulder is in 90°-45° provocative positioning.

### Subpectoral Identification of Long Head of Biceps Tendon

**Fig 3:**
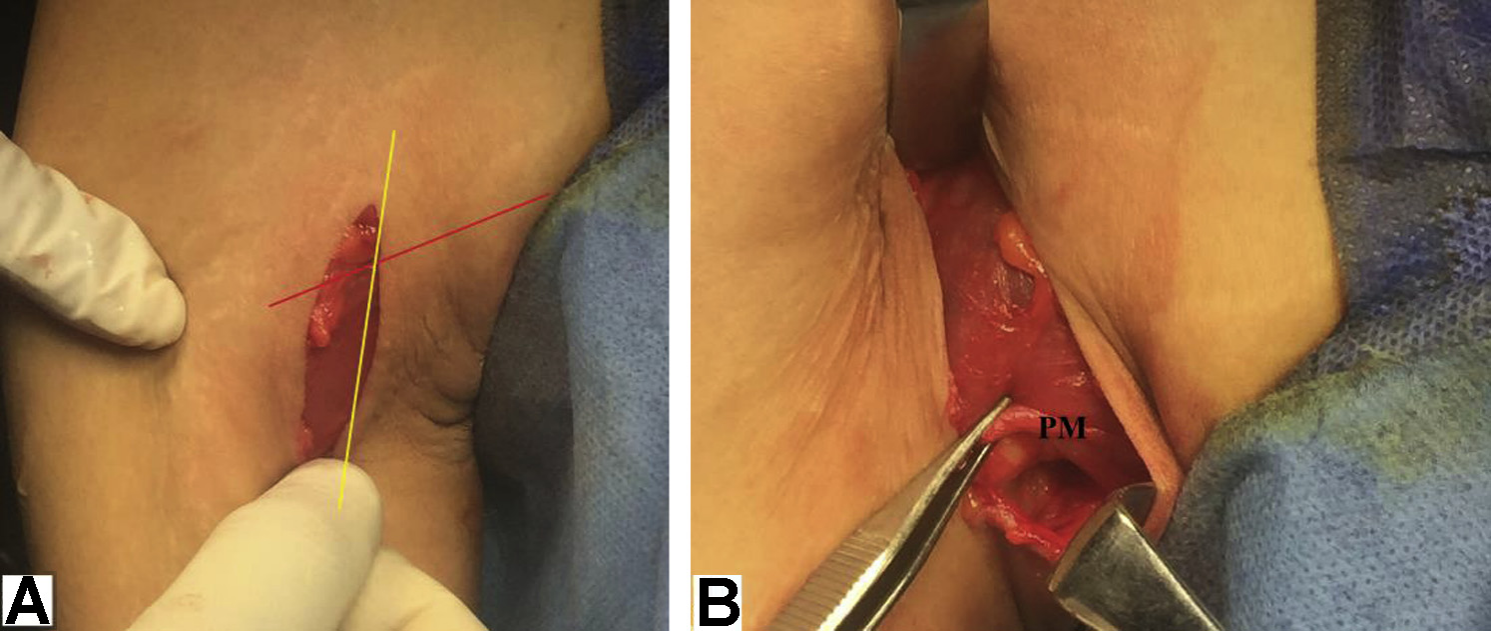
(A and B) Identification of lower border of pectoralis major muscle in the right shoulder while seating the patient in beach-chair position. (A) Through a 5-cm vertical skin incision centered at crossing point of (red line-marked) lower border of PM and (yellow line-marked) antero-medial border of the arm; subcutaneous tissue is dissected to expose the fascia (covering PM). (B) This fascia is then swept off by a gauze sponge to facilitate visualization of lower border of PM pectoralis major; PM, pectoralis major (lower border).

Through a 5-cm vertical skin incision centered at the crossing point of lower border of PM and antero-medial border of the arm; subcutaneous tissue is dissected in line with skin incision to identify the fascia covering PM. This fascia is then swept off by a gauze sponge to help visualize lower border of PM, which is then cautiously retracted superiorly using a soft tissue retractor. Figs 3, A and B, demonstrate identification of lower border of PM. Pearls and pitfalls of the reported technique are summarized in Table 1.

**Table 1:** Pearls and Pitfalls of This Technique

Pearls
• Subpectoral identification of LHB
• Stay/marking absorbable suture of LHB at level of lower border of PM
• Arthroscopic LHB tenotomy from its superior labral origin
• Subpectoral delivery, trimming, and whip-stitching (with a couple of # 2 nonabsorbable sutures) of free end of LHB
• Removal of anterior mid-glenoid cannula
• Establishment of subpectoral tunnel by surgeons’ finger
• Passage of a blunt arthroscopic trocar/sheath deep to lower border of PM to appear through anterior mid-glenoid portal
• Passage of shuttle suture through the arthroscopic sheath to retrieve whip-stitched LHB out of the anterior mid-glenoid portal
• Sharp long straight artery clamp (or sharp arthroscopic trocar)/shaver blade-assisted creation of SSC split at 1 and 2 cm from its upper border and its insertion into lesser tuberosity, respectively
• Passage of whip-stitching sutures through the anterior mid-glenoid cannula (from its articular to diaphragm openings using a ring forceps), which is then placed back into its corresponding portal
• Shuttling of the proximal whip-stitching sutures (using a ring forceps) through SSC split followed by retrieval of these sutures above SSC tendon back out of the anterior mid-glenoid cannula and pulling of these sutures until whip-stitched LHB is driven through SSC split
• Knot tying of proximal and distal whip-stitching sutures, so that LHB is sutured over itself around 1cm cuff of upper SSC tendon
• Securing tenodesis by additional simple stitches (using direct suture passer) to firmly hold LHB and SSC tendon together.
• Testing integrity of soft tissue LHB tenodesis (under arthroscopic visualization) by probing, axial loading of LHB by an artery clamp, and 90°-90° provocative GH positioning
Pitfalls
• To have adequate length of tenotomized LHB to be sutured over itself, free ends of proximal and distal whip-stitching sutures of LHB tendon should be at least 3 cm apart.
• To facilitate suture identification during future knot tying, whip-stitching of LHB tendon should be performed with 2 nonabsorbable sutures of different colors.
• For easy and safe subpectoral corridor during passing the arthroscopic trocar/sheath, the shoulder should be placed in slight extension to facilitate sheath passage. A blunt trocar is used to avoid injury to the axillary nerve, and the sheath should be kept lateral to the coracoid process to avoid injury of the musculo-cutaneous nerve.
• Safe creation of SSC window necessitates splitting of its upper tendon close to its insertion and avoiding use of diathermy probe to avoid axillary nerve injury.
• Effective creation of SSC window necessitates splitting of its tendon at a distance of at least 1 cm from its upper border to offer sufficient cuff of upper SSC for future tenodesis
• To reestablish physiologic biceps length-tension relationship, knot tying of whip-stitching sutures should be performed while keeping marking absorbable sutures at the level of lower border of PM.
• Concurrent procedures (e.g. Bankart repair, remplissage) should be performed following biceps tenodesis; otherwise, repaired structures (antero-inferior labrum, ISP tendon) might be subjected to excessive tension (while performing tenodesis/testing tenodesis integrity) leading to repair failure.

ISP, infraspinatus; LHB, long head of biceps brachii; PM, pectoralis major; SSC, subscapularis.

**Fig 4:**
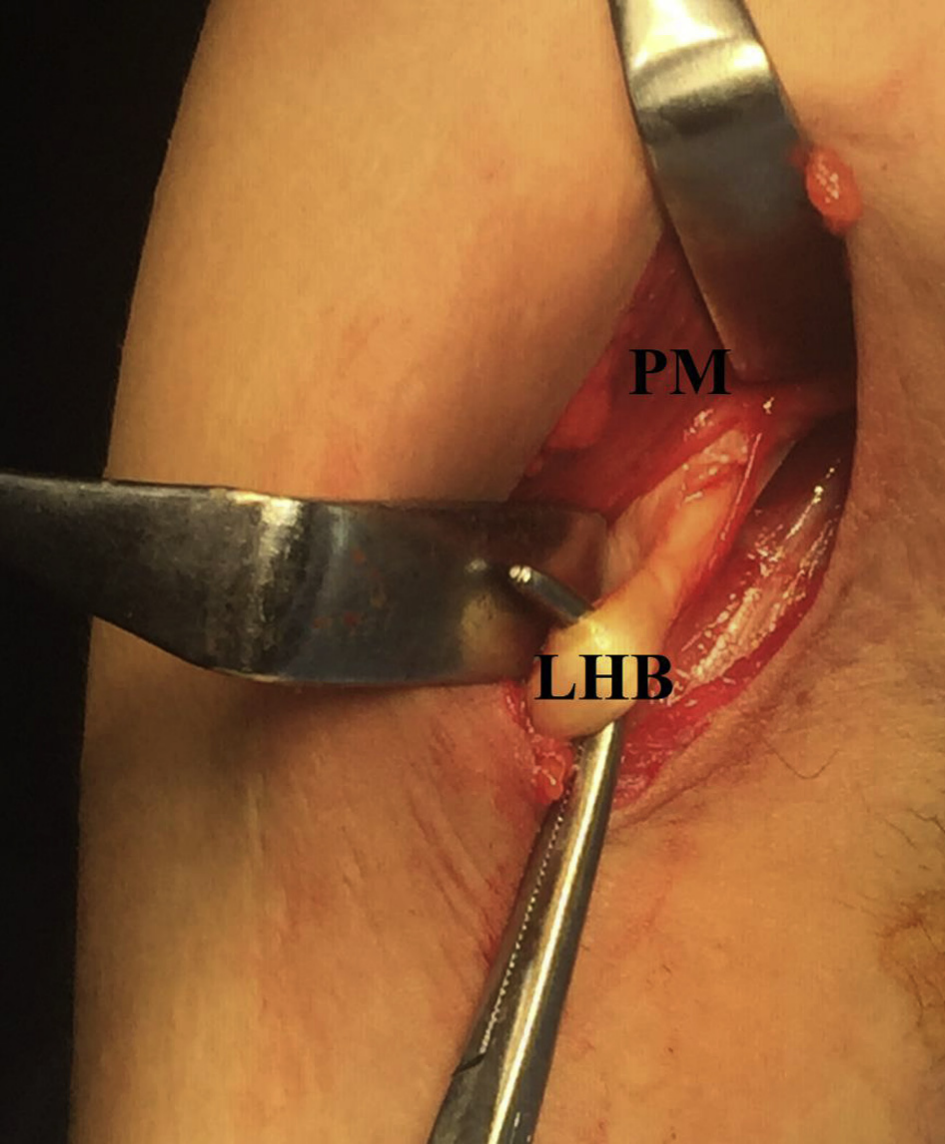
Subpectoral identification of long head of biceps tendon in the right shoulder while seating the patient in beach-chair position. Antero-medial side of the upper arm is explored by blunt dissection until identification of LHB tendon, which can be readily rolled by surgeon’s fingertip against the proximal humerus. LHB, long head of biceps tendon; PM, pectoralis major (lower border).

By blunt dissection, antero-medial side of the upper arm is explored until identification of LHB tendon, which can be readily rolled by surgeon’s fingertip against the proximal humerus. Then, LHB tendon is lifted up over a curved artery clamp, cleared off surrounding soft tissues, and stitched at the level of lower border of PM by # 2 absorbable suture (Vicryl, Ethicon, Cincinnati, OH). The latter suture is to function as a stay suture (i.e., prevents uncontrolled escape of LHB tendon into the arm following tenotomy), and as a marking suture for future reestablishment of biceps muscle length-tension relationship. Fig 4 shows subpectoral identification of LHB tendon.

### Tenotomy of Long Head of Biceps Tendon

**Fig 5:**
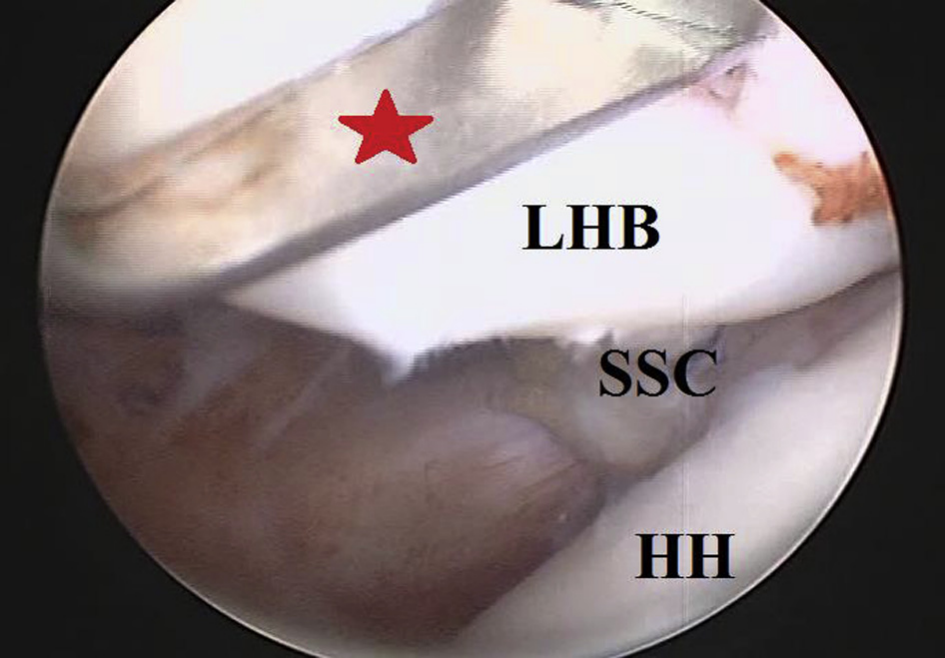
Arthroscopic tenotomy of long head of biceps tendon from its superior labral origin in the right shoulder while seating the patient in beach-chair position, arthroscopically-viewing from the standard posterior gleno-humeral portal and working through the anterior mid-glenoid portal. Long head of biceps is tenotomized from its superior labral origin by combination of (red star-marked) soft tissue biter and motorized shaver blade. HH, humeral head; LHB, long head of biceps tendon; SSC, subscapularis (upper border of its tendon).

Under arthroscopic visualization from the posterior GH portal and through the anterior mid-glenoid portal, tenotomy of LHB from its superior labral origin is performed using a combination of soft tissue biter and motorized shaver blade. Fig 5 shows arthroscopic tenotomy of LHB tendon from its superior labral origin.

### Whip-Stitching of Long Head of Biceps Tendon

**Fig. 6:**
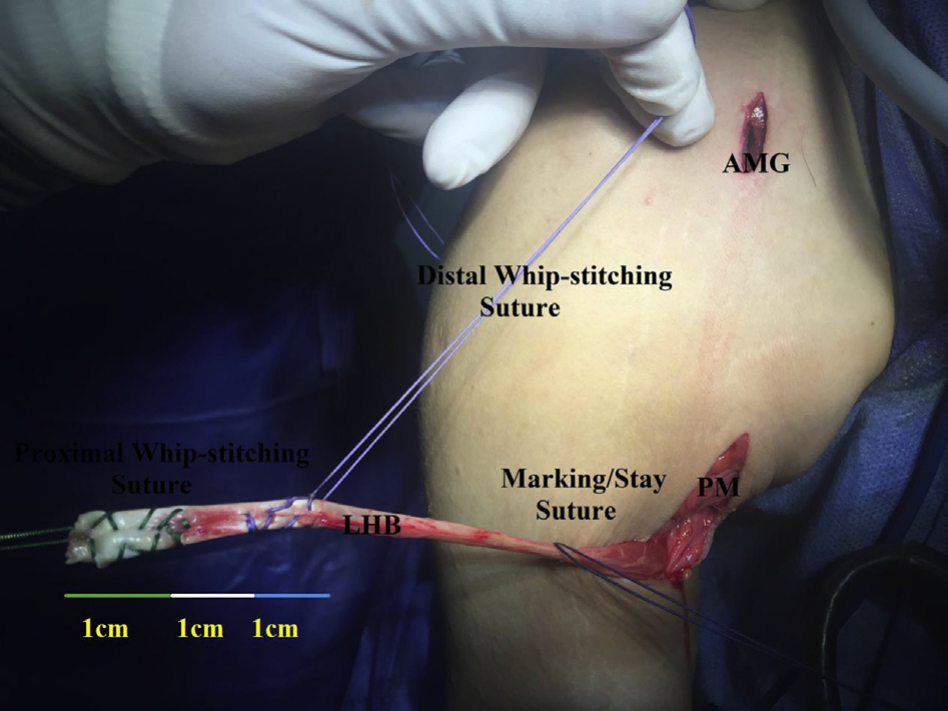
Whip-stitching of free end of long head of biceps tendon in the right shoulder while seating the patient in beach-chair position. Free end of long head of biceps tendon is trimmed off and whip-stitched by a couple of # 2 nonabsorbable sutures of different colors over a length of 1 cm for each suture with an intervening whip-stitch-free distance of 1 cm; whip-stitching should be performed in a way that free ends of the sutures are kept 3 cm apart; AMG, anterior mid-glenoid portal; LHB, long head of biceps tendon; PM, pectoralis major (lower border).

Then, LHB tendon is pulled out (aided by the stay suture) from under the cover of PM. Free end of LHB tendon is trimmed off and whip-stitched by a couple of # 2 nonabsorbable sutures of different colors (Ethibond∗Excel, Ethicon, Cincinnati, OH, / FiberWire suture, Naples, FL) over a length of 1 cm for each suture with an intervening whip-stitch-free distance of 1 cm. Whip-stitching should be performed in a way that free ends of the sutures are kept 3 cm apart. Fig 6 shows whip-stitching of free end of LHB tendon.

### Rerouting of Long Head of Biceps Tendon

**Fig 7:**
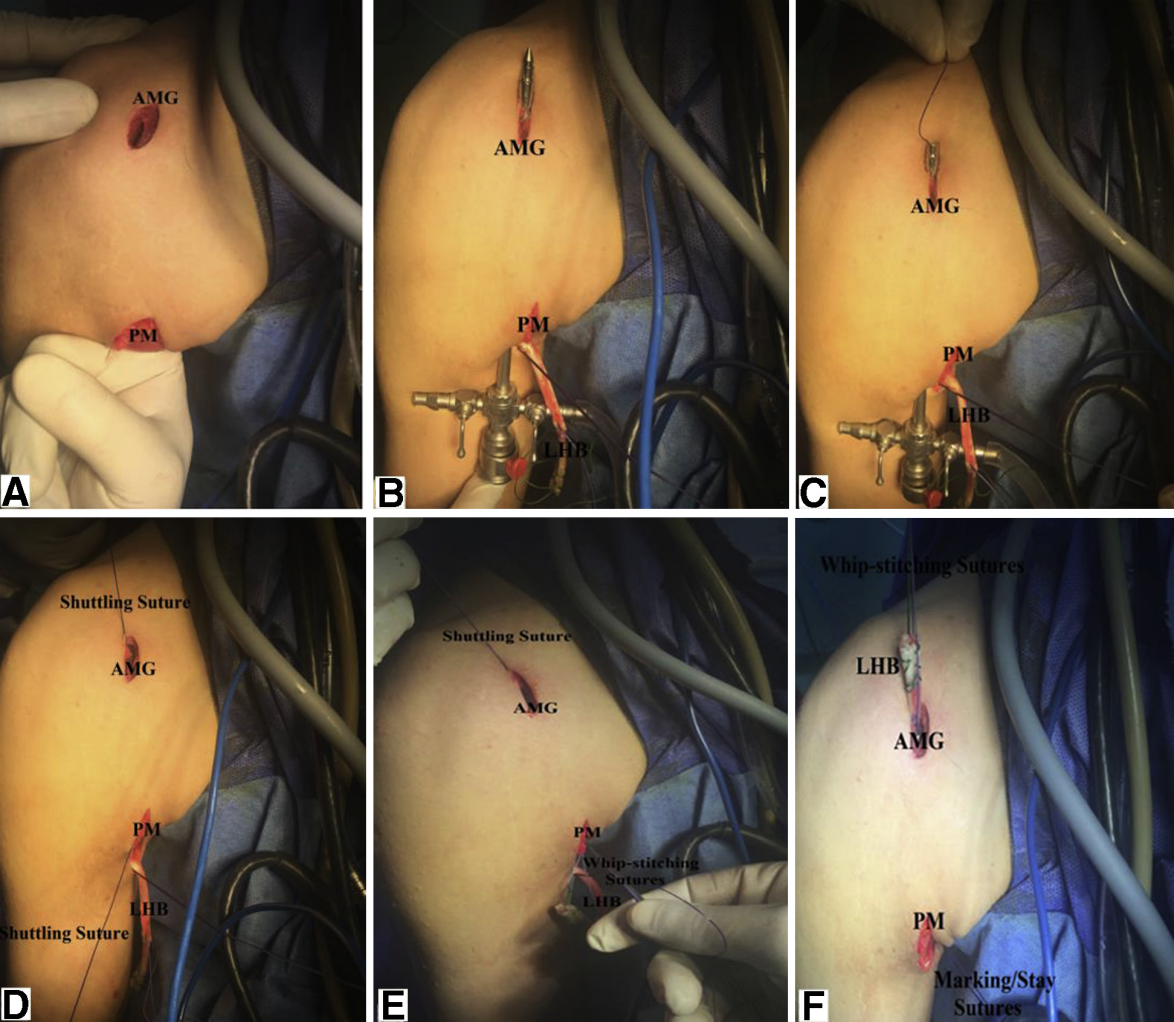
(A-F) Shuttling of whip-stitched long head of biceps tendon out of anterior mid-glenoid portal in the right shoulder while seating the patient in beach-chair position. (A) After removal of anterior mid-glenoid cannula, index finger is passed deep to PM toward anterior mid-glenoid portal to establish a sub-pectoral track. (B) A blunt arthroscopic trocar/sheath are passed deep to lower border of PM to appear through the anterior mid-glenoid portal. (C) Arthroscopic trocar is removed from its sheath through which a shuttling suture is passed to appear deep to PM. (D) Arthroscopic sheath is removed. (E and F) Shutting suture is tied to whip-stitching sutures and pulled out of the anterior mid-glenoid portal in order to deliver LHB tendon out of anterior mid-glenoid portal. AMG, anterior mid-glenoid portal; LHB, long head of biceps tendon; PM, pectoralis major (lower border).

Anterior mid-glenoid cannula is then removed to provide a room for a blunt arthroscopic trocar/sheath to be passed deep to lower border of PM to appear through the anterior mid-glenoid portal in order to deliver whip-stitched LHB tendon (using shuttle sutures passed through the arthroscopic sheath) out of anterior mid-glenoid portal. Fig 7, A-F, show shuttling of whip-stitched LHB tendon out of anterior mid-glenoid portal.

### Subscapularis Window

**Fig 8:**
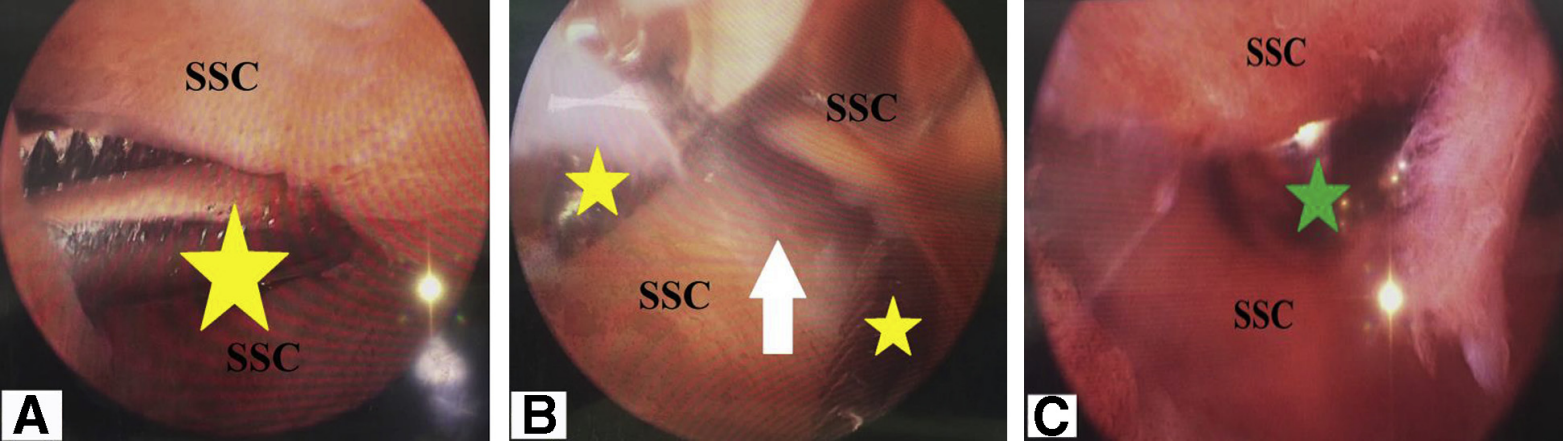
Creation of subscapularis window in the right shoulder while seating the patient in beach-chair position, arthroscopically-viewing from the standard posterior gleno-humeral portal and working through the anterior mid-glenoid portal. (A) A yellow star shows the sharp long straight artery clamp (or alternatively a sharp arthroscopic trocar) that is used to pierce SSC tendon at a distance of 1 cm from its upper border and of about 2 cm from its insertion into the lesser tuberosity. (B) Yellow stars show that the straight artery clamp is repeatedly opened/closed to create (white arrow-marked) SSC hole. (C) A green star marks where the motorized shaver blade is used to widen SSC hole into a window/split of about 1-1.5-cm in length. SSC, subscapularis tendon.

Thereafter, at a distance of 1 cm from its upper border and of about 2 cm from its insertion into the lesser tuberosity; SSC tendon is pierced (under arthroscopic visualization from the posterior GH portal) by a sharp long straight artery clamp (or alternatively by a sharp arthroscopic trocar); creating a SSC hole that can be widened by motorized shaver blade into a SSC window/split of about 1-1.5 cm in length. Fig 8, A-C, show creation of SSC window.

### Passage of Long Head of Biceps Tendon Within Subscapularis Window

Afterward, an arthroscopic ring forceps is used to pass whip-stitching sutures through the anterior mid-glenoid cannula (from its articular to diaphragm openings), which, in turn, is placed back into its corresponding portal.

**Fig 9:**
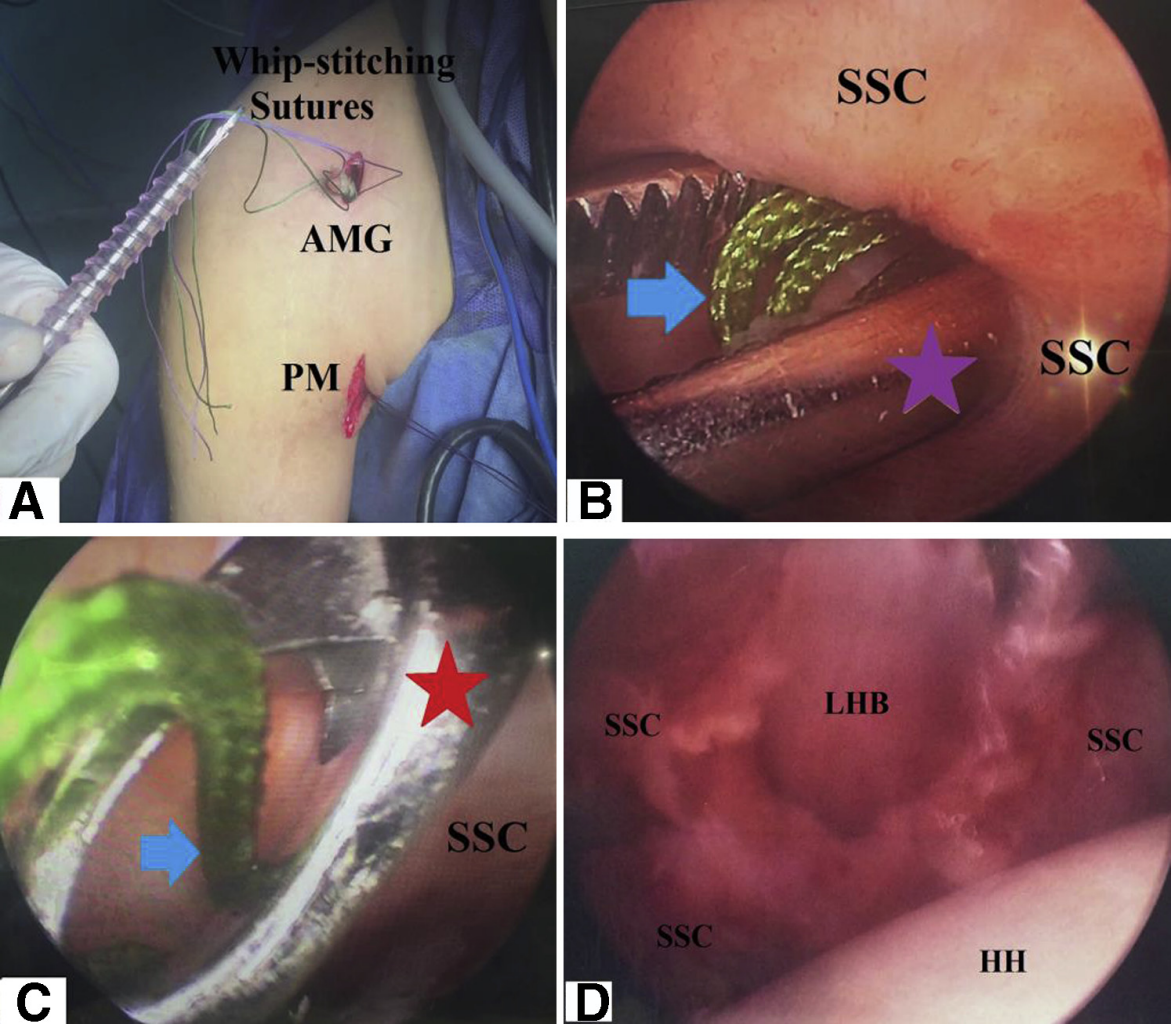
Passage of whip-stitched long head of biceps tendon through subscapularis window in the right shoulder while seating the patient in beach-chair position, arthroscopically viewing from the standard posterior gleno-humeral portal and working through the anterior mid-glenoid portal. (A) Arthroscopic ring forceps is used to pass whip-stitching sutures through the anterior mid-glenoid cannula (from its articular to diaphragm openings), which, in turn, is placed back into its corresponding portal. (B) Using (violet star-marked) arthroscopic suture grasper (or alternatively an arthroscopic ring forceps), free ends of (blue arrow-marked) proximal whip-stitching sutures are passed through SSC window to be delivered behind SSC. (C) A red star marks where arthroscopic ring forceps is used to retrieve (blue arrow-marked) delivered proximal whip-stitching sutures above SSC and then out of the anterior mid-glenoid cannula. (D) Retrieved sutures are pulled until whip-stitched LHB tendon is shuttled through SSC window into the gleno-humeral joint. AMG, anterior mid-glenoid portal; HH, humeral head; LHB, long head of biceps tendon; PM, pectoralis major (lower border); SSC, subscapularis tendon.

Using an arthroscopic ring forceps (or suture grasper), free ends of the proximal whip-stitching sutures are passed through SSC window to be delivered behind SSC. An arthroscopic ring forceps is then used to retrieve the delivered sutures above SSC tendon and then out of the anterior mid-glenoid cannula. Retrieved sutures are pulled until whip-stitched LHB tendon is shuttled through SSC window into GH joint. Fig 9, A-D, show passage of whip-stitched LHB tendon through SSC window.

### Tenodesis of Long Head of Biceps to Subscapularis Tendon

**Fig. 10:**
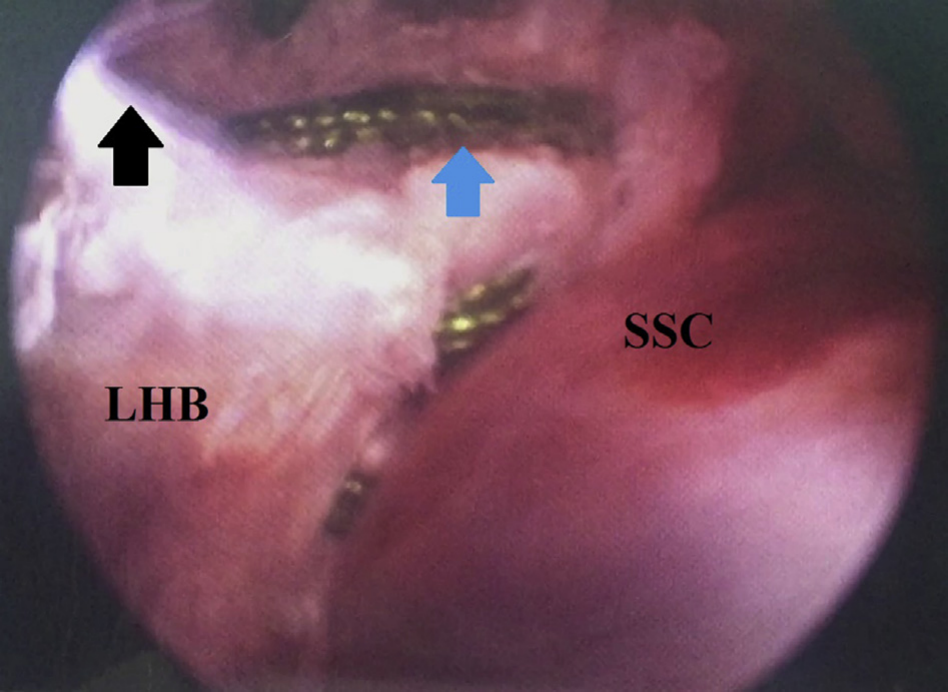
Knot tying of tenodesis of long head of biceps to upper subscapularis in the right shoulder, while seating the patient in beach-chair position, arthroscopically viewing from the standard posterior gleno-humeral portal and working through the anterior mid-glenoid portal. (Blue arrow-marked) retrieved proximal whip-stitching suture is tied with its (black arrow-marked) distal counterpart thus, whip-stitched end of LHB tendon is sutured over itself around a 1-cm cuff of upper border of SSC tendon. LHB, long head of biceps tendon; SSC, subscapularis upper border of its tendon.

Next, a limb of retrieved proximal whip-stitching sutures is tied to a limb of the distal whip-stitching sutures, and the knot is pushed down the cannula by a knot pusher until resting on LHB tendon. This knot is further secured by 6 alternating half hitches. The remaining untied sutures are then tied in a similar fashion. Knot tying eventually results in suturing whip-stitched end of LHB tendon over itself around a 1-cm cuff of upper border of SSC tendon. Fig 10 shows knot tying of proximal and distal whip-stitching sutures of LHB tendon.

**Fig. 11:**
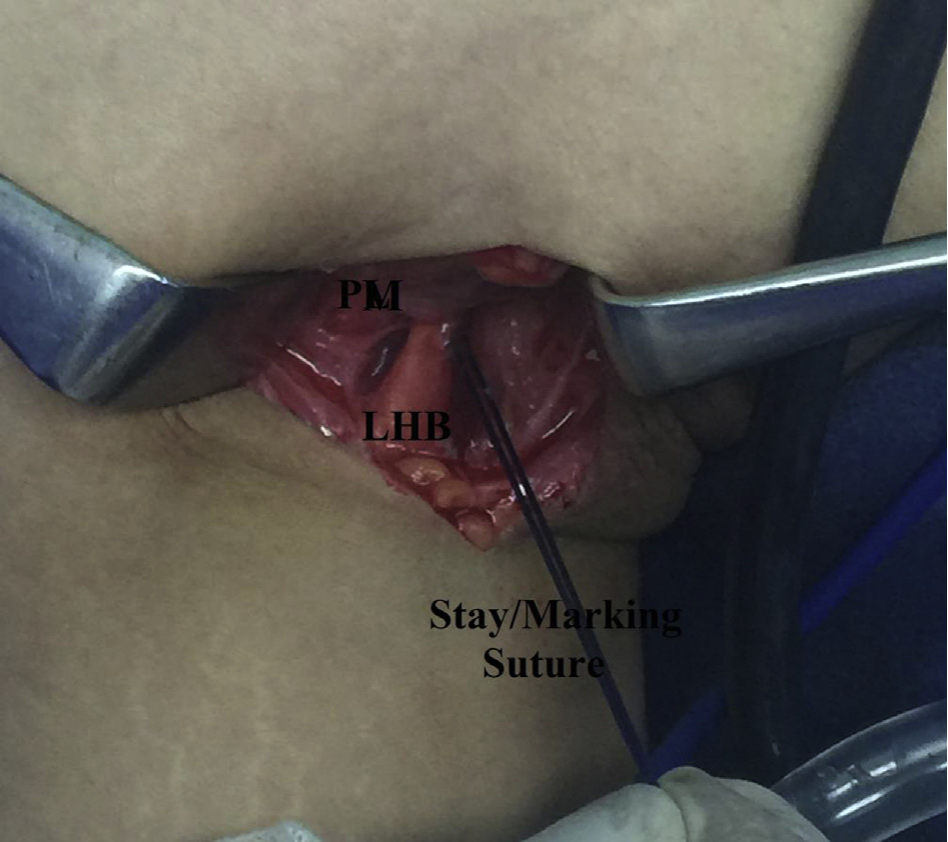
Leveled marking suture of long head of biceps tendon at lower border of pectoralis major during knot tying of whip-stitched long head of biceps tendon over itself in the right shoulder, while seating the patient in beach-chair position. This leveling helps re-establish biceps muscle length-tension relationship. LHB, long head of biceps tendon; PM, pectoralis major (lower border).

During knot tying, marking absorbable suture should be kept leveled with lower border of PM for accurate reestablishment of biceps muscle length-tension relationship. Fig 11 demonstrates leveling of marking absorbable suture of LHB tendon at lower border of PM during knot tying of LHB tendon.

**Fig 12:**
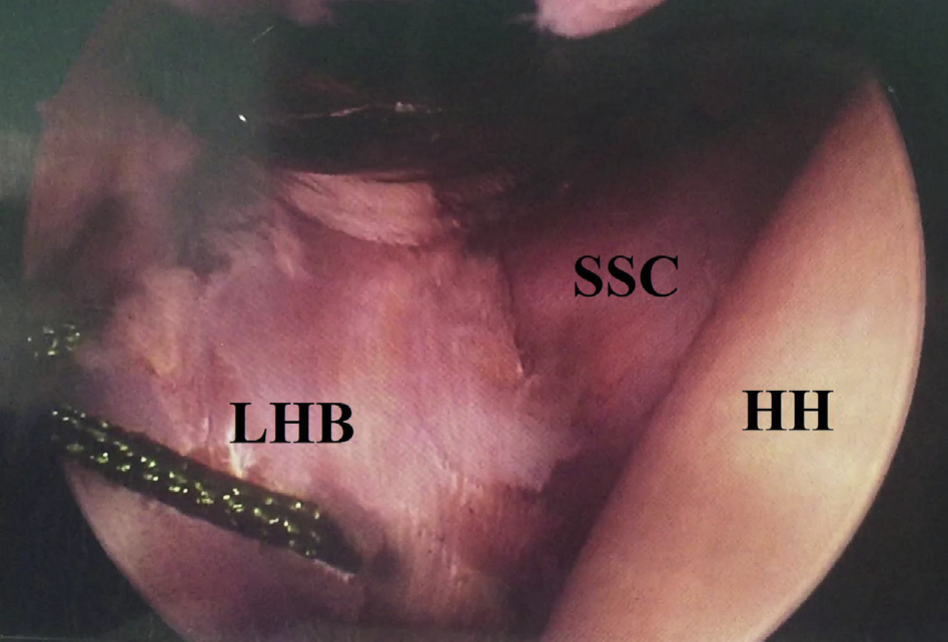
Picture of completed extra-articular soft arthroscopic Latarjet technique (ex-SALT) in the right shoulder while seating the patient in beach-chair position, arthroscopically viewing from the standard posterior gleno-humeral portal and working through the anterior mid-glenoid portal. HH, humeral head; LHB, long head of biceps tendon; SSC, subscapularis (upper border of its tendon).

This tenodesis can be further secured by additional simple stitches (using a direct suture passer) to firmly hold LHB and SSC tendons together. Fig 12 shows a picture of completed currently reported technique.

### Dynamic Testing of the Construct

**Fig 13:**
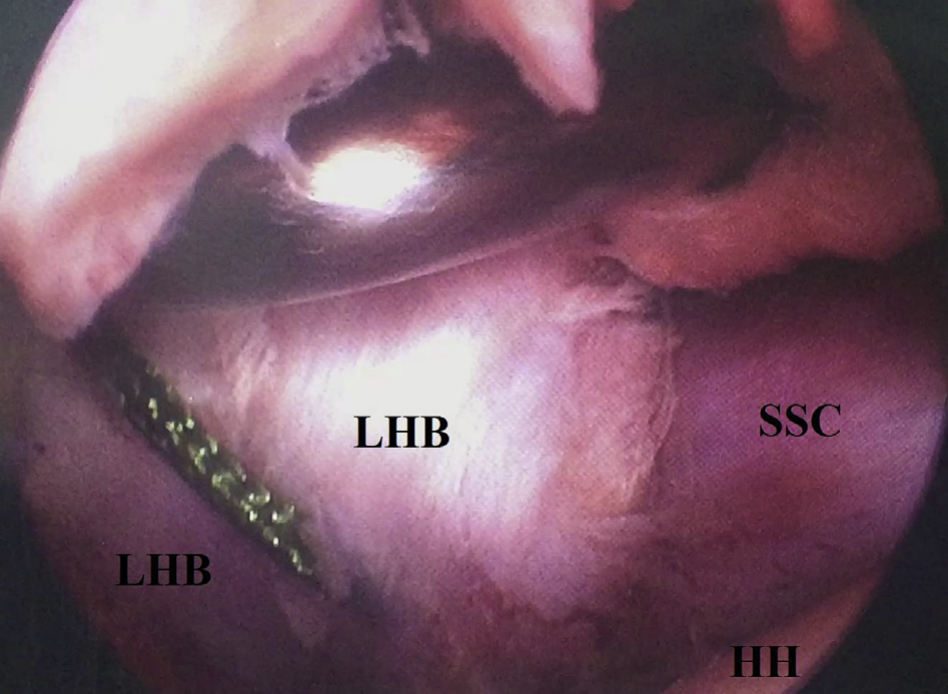
The right shoulder is probed to test the integrity of tenodesis of long head of biceps to upper subscapularis while the patient is seated in a beach-chair position. Shown is an arthroscopic view from the standard posterior gleno-humeral portal as the surgeon works through the anterior mid-glenoid portal. HH, humeral head; LHB, long head of biceps tendon; SSC, subscapularis upper border of its tendon.

Under arthroscopic visualization from the posterior GH portal, integrity of soft tissue tenodesis of LHB to SSC tendon is checked by probing, axial loading of LHB tendon by an artery clamp, and provocative 90°-90° GH positioning. The latter 2 maneuvers are helpful in exhibiting hammock-mechanism of SSC in restoration of GH stability. Fig 13 shows tested integrity of tenodesis of LHB to upper SSC tendon by probing.

For more detailed illustration, technical steps of currently reported ex-SALT are shown in Video 1.

### Standard Bankart Repair

Following ex-SALT, standard arthroscopic Bankart repair is performed; including freeing, mobilization, and reduction of detached capsule-labral complex, decortication of antero-inferior glenoid bed, and suture anchor repair using 3 single-loaded anchors (1.4 mm JuggerKnot All-Suture Anchors, Zimmer Biomet, Warsaw, IN) implanted at 5, 4, and then 3 o’clock positions. A direct suture passer (Zimmer Biomet, Warsaw, IN) is used to deliver one or both suture limbs for either simple or mattress suturing of the capsule-labral complex, respectively.

### Postoperative Rehabilitation

Operated shoulder is immobilized in a sling for 6 weeks. However, during this period, 90°-150° active ROM of the elbow and isometric deltoid exercises are encouraged. Thereafter, sling is discarded, light daily-living activities are allowed, and pendulum and assisted-active ROM exercises are practiced for 2 weeks. By 8 weeks postoperatively, physiotherapy rehabilitation protocol is commenced for 2 weeks of stretching ROM exercises followed by 4-6 weeks of active and strengthening exercises. Resisted elbow flexion and return to overhead and sports activities are permitted by 16-20 weeks postoperatively.

## Discussion

Recently, increasing numbers of technical and biomechanical reports on gaining advantage of LHB tendon for management of anterior GH instability have been published.^9^, ^10^, ^11^, ^12^, ^13^, ^14^

Initially in 2017, Tang et al. described arthroscopic trans-subscapularis transfer of LHB tendon and its fixation into the anterior glenoid within a retrograde-drilled tunnel by a suspension device. Later in 2018, Collin et al. reported a technical modification of the preceding description in which LHB tendon is fixed by a tenodesis screw and coined the term dynamic anterior stabilization (DAS) of the shoulder to refer to this currently evolving GH reconstructive concept. Furthermore, in 2019, Milenin et al. published a closely related technique, in which LHB can be used for concurrent DAS and labral reconstruction.^9^, ^10^, ^11^

In addition, different biomechanical studies showed DAS effectiveness in restraining GH translation in the setting of subcritical (<20%) anterior glenoid bone loss.^13^^,^^14^

However, reported DAS techniques might herald relative disadvantages of technical complexity, long operative time, slow learning curve, and implant-related cost. For simplification, Kandeel described intra-articular soft arthroscopic Latarjet technique (in-SALT) in which Bankart repair is followed by tenodesis of LHB tendon to upper SSC by 2 simple stitches of nonabsorbable sutures. However, this latter technique does not closely reproduce stabilizing mechanisms of Latarjet procedure.^12^

For more technical simplification and closer reproducibility of sling and hammock mechanisms of Latarjet procedure, the current article reports on extra-articular soft arthroscopic Latarjet technique (ex-SALT), referring to intra-articular tenotomy, subpectoral delivery, whip-stitching and re-routing and passage through SSC window of LHB, which is then sutured over itself around upper border of SSC tendon.

### Indications and Contraindications

The currently reported technique can be applied for management of wide spectrum of capsule-labral detachment/deficiency of anterior GH instability. Notably, it is crucial to clarify that these versatile indications include management of high-risk patients for postoperative recurrence of GH instability (i.e., overhead athletes and patients with generalized joint laxity or with multidirectional GH instability of antero-inferior predominance) and revision GH restabilization procedures. In addition, repair of rotator cuff following elderly GH dislocation can be coupled with ex-SALT instead of currently exercised biceps tenodesis techniques. Indications and contraindications of the reported technique are summarized in Table 2.

**Table 2:** Indications And Contraindications of This Technique

Indications
Type-V SLAP lesion
Deficient antero-inferior capsulo-labral complex
Concomitant LHB lesions (i.e., tendinitis, <30% tear, instability, and pulley lesion)
High-risk patients for postoperative recurrence of GH instability (hyperlaxity, contact/competitive/overhead sports activity)
Multidirectional instability with antero-inferior predominance
In conjunction with repair of rotator cuff tear on top of acute GH dislocation in the elderly
Revision management of GH instability
Contraindications
Critical deficiency (>20% bone loss) of the anterior glenoid
Engaging Hill-Sachs lesions
Extensive intra-articular LHB lesion involving the proposed tendon segment for whip-stitching and tenodesis (relative)

GH, gleno-humeral; LHB, long head of biceps brachii; SLAP, superior labrum from anterior to posterior.

### Technical Considerations

Compared with described DAS techniques, ex-SALT is simpler and quicker, as it depends upon tenodesis of rerouted LHB tendon to upper SSC (i.e., no implants for glenoid bony fixation). A comparison of ex-SALT, in-SALT, and DAS technique of Collin et al. is summarized in Table 3.^10^^,^^12^

**Table 3:** Technical Differences of This Technique From That of Its Intra-Articular Counterpart and From That of Dynamic Anterior Stabilization of Collin et al.

Technical Difference	ex-SALT Technique	in-SALT Technique	DAS Technique
Decubitus	Beach chair	Beach chair	Beach chair
Viewing Portals	Posterior	Posterior	Posterior Antero-supero-lateral
Working Portals	Anterior mid-glenoid	Anterior mid-glenoid Antero-supero-lateral	Anterior mid-glenoid Posterior
Working spaces	GH Subpectoral	GH	GH Subcoracoid
Rotator interval	Not released	Not released	widely opened
Bicipital sheath	Not opened	Not opened	Opened
Location of SSC split	1 cm inferior and 2 cm medial to upper border and humeral insertion of SSC, respectively	No split	3-3.5 cm from upper border of SSC
Creation of SSC split	Artery clamp and shaver blade		Retrograde suture passer and external GH rotation
Relation of rerouted LHB to CT	Lateral	Lateral	Posterior
Reduction of diameter of proximal LHB	Not needed	Not needed	Needed to adapt to glenoid tunnel
Labral traction suture	Not needed	Not needed	Needed to maximize visualization field for anterior glenoid drilling
Site of tendon fixation	Within upper SSC window	Upper SSC tendon	Anterior glenoid
Method of fixation	Suturing whip-stitched tenotomized LHB stump on itself around a cuff of upper SSC tendon, with further augmentation of this tenodesis by simple stitches	2 simple stitches holding tenotomized LHB and upper SSC tendons together	Knotless anchor within a glenoid tunnel
Knot tying	Over LHB	Over LHB	
Tenotomy	Prior to tenodesis	Following tenodesis	Prior to tenodesis
Biceps length-tension relationship	Reestablished		

DAS, dynamic anterior stabilization; ex-SALT LHB, extra-articular soft arthroscopic Latarjet technique; GH, gleno-humeral; in-SALT, intra-articular soft arthroscopic Latarjet technique; LHB, long head of biceps brachii; SSC, subscapularis.

Since the first descriptions of trans-subscapularis transfer of conjoint tendon of Boileau et al. and of osteotomized coracoid graft of Lafosse et al., creation of SSC window/split was to some extent technically challenging because of relatively large size of the transferred structures. Regarding the LHB tendon, it has a relatively smaller cross-sectional area, which eases its trans-subscapularis transfer. This transfer can, in turn, be in turn, be further facilitated by reduction of diameter of proximal LHB tendon during trimming.^1^, ^2^, ^3^, ^4^, ^5^, ^6^, ^7^, ^8^^,^^10^^,^^15^^,^^16^

Furthermore, different maneuvers have been exercised to simplify creation of SSC split and passage of LHB tendon, such as moving the shoulder alternately in external and internal rotation after passage of the whip-stitching sutures through the SSC, so that these sutures can spread SSC along its fibers.^16^

In ex-SALT, this is not the case, as LHB tendon was shuttled through artery clamp/shaver blade-created SSC window. In addition, instead of rotating the shoulder; whip-stitching sutures can be tensioned (while shuttling LHB tendon) and repeatedly moved medio-laterally to widen SSC window when needed.

It is worth mentioning that, ex-SALT permits creation of SSC window in advance to reinsertion of anterior mid-glenoid cannula; this advantage eases instrumental maneuvering during window creation and protects whip-stitching sutures by keeping them away from working instruments.

Lately, Garcia et al. described a modified DAS technique performed through 2 working portals, including 1-antero-infero-lateral GH portal (created 1 cm inferior and just lateral to the standard anterior mid-glenoid portal); and 2- antero-lateral sub-acromial/sub-deltoid portal (for visualization, while pulling tenotomized LHB tendon out of the bicipital groove just proximal to upper border of PM and for release of bicipital tunnel when needed). Meanwhile, the currently reported technique does not necessitate special or additional working portals.^17^

Contrary to reported DAS techniques, accurate restoration of biceps muscle length-tension relationship is feasible during ex-SALT as knot tying of shuttled LHB tendon can be adjusted according to level of the initial marking absorbable sutures with respect to lower border of PM. For that reason, tenotomy of LHB tendon is delayed until it is identified and suture-marked in the subpectoral region.

### Biomechanical Considerations

**Fig 14:**
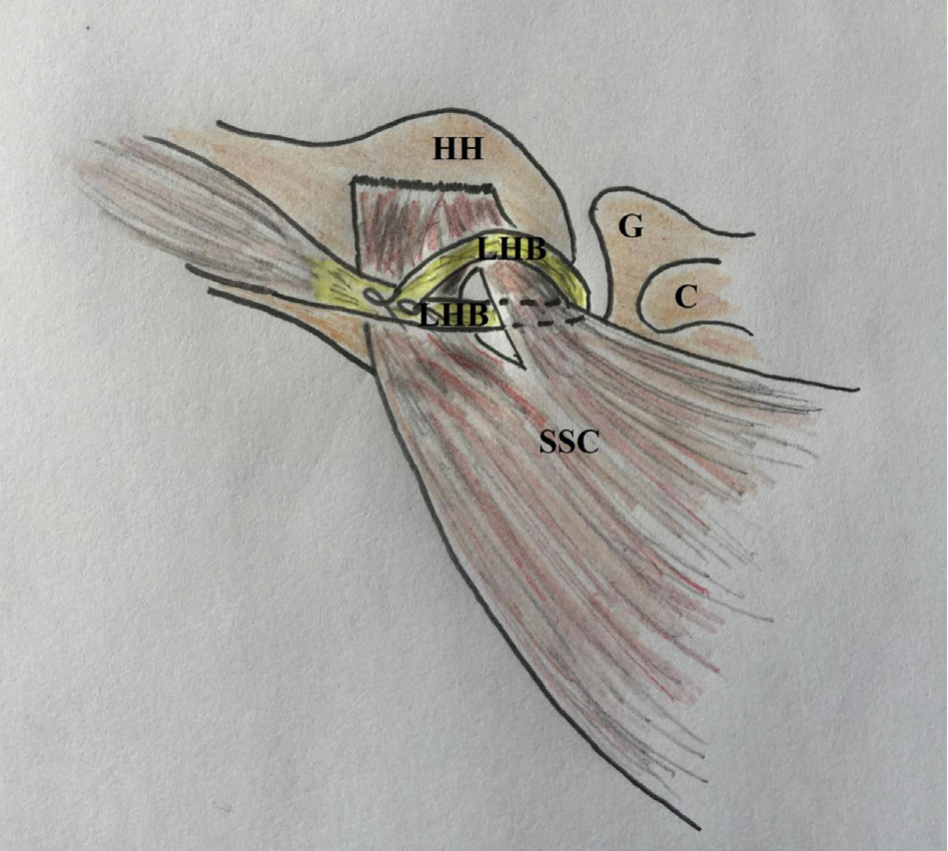
Sling and hammock gleno-humeral restabilization mechanisms of the extra-articular soft arthroscopic Latarjet technique (ex-SALT) are shown in the right shoulder. C, coracoid process; G, glenoid; HH, humeral head; LHB, long head of biceps tendon; SSC, subscapularis muscle.

Compared to its intra-articular counterpart, ex-SALT more effectively reproduces both sling and hammock GH restabilization mechanisms of Latarjet procedure. Fig 14 shows sling and hammock gleno-humeral restabilization mechanisms of the currently reported technique. In addition to 2 preceding dynamic mechanisms, further restoration of GH stability can be achieved via feasible concurrent capsule-labral repair (static mechanism) following ex-SALT. However, ex-SALT does not reconstruct the bone-deficient anterior glenoid.

Accordingly, ex-SALT offers 3 out 4 of GH restabilization mechanisms of the Latarjet procedure. A comparison of GH restabilization mechanisms offered by ex-SALT, in-SALT, and Latarjet procedure is summarized in Table 4.^12^^,^^18^

**Table 4:** Comparison of Gleno-humeral Restabilization Mechanisms of Currently Reported Technique, Its Intra-articular Counterpart, and Latarjet Procedure

Mechanism	ex-SALT Technique	in-SALT Technique	Latarjet Procedure
Sling Mechanism	++ (LHB)	+ (LHB)	+++ (CT)
Hammock mechanism of SSC	+++	+ (Curtain-like effect rather than hammock mechanism)	+++
Concurrent capsulo-labral repair	+	+	+
Restoration of bony glenoid articular arc and concavity	_	_	+

CT, conjoint tendon; ex-SALT LHB, extra-articular soft arthroscopic Latarjet technique; in-SALT, intra-articular soft arthroscopic Latarjet technique; LHB, long head of biceps brachii; SSC, subscapularis. The symbols (+/-) refer to how much each gleno-humeral restabilization mechanism is offered by the compared techniques.

Currently evolving techniques of DAS and SALT are soft tissue GH reconstructive procedures that do not address glenoid bony deficiency; however, this does not raise major concerns, as these techniques are used for GH instability with subcritical bone defects. Besides, a biomechanical study of Yamamoto et al. investigating GH restabilization mechanisms of Latarjet procedure demonstrated that at provocative positions of GH instability (i.e., high angles of abduction-external rotation); GH stability is offered mainly by sling and hammock mechanisms (77%) rather than by capsule-labral reconstruction (23%); meanwhile, bony glenoid reconstruction by coracoid graft reaches its peak contribution (45%) in GH restabilization only at lower angles of abduction.^18^

In a recent biomechanical study, Mehl et al. demonstrated effectiveness of DAS technique in restraining GH translation in unstable shoulders with subcritical (<20%) anterior glenoid bone loss and pointed out that DAS techniques are to bridge the gap between indications of soft tissue and bony GH reconstructive techniques.^13^

Furthermore, in a cadaveric model of 12 tested shoulders of subcritical (<13%) glenoid bone loss, Bokshan et al. concluded that Bankart repair when coupled with DAS technique had greater peak resistance force to anterior translation and was significantly stronger (by 16.6%) than when coupled with conjoint tendon transfer (i.e., Bristow procedure), recommending DAS augmentation of Bankart repair in high-risk patients (i.e., for postoperative recurrence of GH instability) with subcritical bone loss.^14^

### Biological Considerations

From another perspective, ex-SALT might have a biological advantage of faster and more consistent healing of tenodesis, as LHB tendon-SSC tendon interface might be superior in its biological characteristics and behavior with subsequent better biomechanical properties than its counterpart of LHB tendon-glenoid bone interface of DAS techniques.^19^

### Potential Advantages

The current description offers the advantage of technical simplicity, as it does not require special arthroscopic training, and it has a steep learning curve due to familiarity of most of shoulder surgeons with subpectoral tenodesis.

Additionally, SSC window needed in ex-SALT is relatively small and is created in a safe location; hence, minimizing risk of SSC damage and injury of nearby neurovascular structures.

Another technical advantage of ex-SALT is that unlike current DAS techniques, it does not violate the subacromial, subdeltoid, and subcoracoid spaces, and it does not necessitate release of the bicipital tunnel (to ensure free motion of rerouted LHB tendon); therefore, the rotator cable and SSC insertion are preserved.^9^, ^10^, ^11^^,^^16^^,^^17^

Furthermore, it is the one working-portal procedure that does not require shifting the scope between the working and accessory portals.

In ex-SALT, LHB tendon does not occupy GH joint; as a result, there is still capacious intra-articular room for easy maneuvering during concurrent procedures, as subsequent Bankart repair and remplissage capsulo-tenodesis. Besides, performing LHB tenodesis prior to these concurrent procedures allows safe global dynamic testing of integrity of this tenodesis.

In addition, as it is a soft tissue procedure; ex-SALT avoids coracoid graft, glenoid, and hardware-related complications of classic Latarjet and DAS techniques.^1^, ^2^, ^3^, ^4^, ^5^, ^6^, ^7^, ^8^, ^9^, ^10^, ^11^^,^^16^^,^^17^

It is needless to point out that revision instability procedures on top of failed ex-SALT are feasible and easy, as both coracoid process and glenoid neck are still virgin.

### Technical Limitations

Regarding technical limitations, ex-SALT is a nonanatomic reconstructive technique; however, this limitation might be overcome by feasible concurrent Bankart repair. Failure of tenodesis is still a risk, with possible recurrence of GH instability and development of cosmetic disfigurement of Popeye sign in such a young, active patient group. Advantages and disadvantages of the reported technique are summarized in Table 5.

**Table 5:** Advantages and Disadvantages of Currently Reported Technique

Advantages
• Sling mechanism
• Hammock mechanism
• Versatile indications
• Technical simplicity, familiarity, quickness, safety, and reproducibility
• Arthroscopic (minimally invasive) procedure
• Intra-articular tenodesis, so it offers a relatively long lever arm of biceps
• Feasible reestablishment of biceps muscle length-tension relationship
• No violation of subacromial or subcoracoid spaces
• No opening of the bicipital groove
• Cost-saving (no hardware)
• Feasible concurrent GH restabilization procedures (e.g., Bankart repair)
• No loss of external rotation (LHB is not fixed into glenoid)
• Fast healing rate of LHB tenodesis (tendon-to-tendon interface)
• Avoidance of bone block and hardware-related complications
• No reoperation for hardware removal
• Relatively easy revision
Limitations
• Nonanatomic
• Small cross-sectional area and proximal location of LHB sling
• No glenoid bony reconstruction
• Technical irreproducibility in extensive LHB lesions
• Questionable postoperative biceps cramping pain
• Possible Popeye deformity
• No biomechanical validation
• No long-term cohort clinical studies

GH, gleno-humeral; LHB, long head of biceps brachii.

### Conclusion

Currently reported technique of extra-articular rerouting and soft tissue tenodesis of tenotomized long head of biceps tendon within a subscapularis window by nonabsorbable sutures provides the advantages of versatility for management of a wide spectrum of different gleno-humeral instability situations, close reproducibility of Latarjet dynamic gleno-humeral restabilization mechanisms, and technical simplicity and familiarity. Nevertheless, further biomechanical and clinical studies are needed to validate its long-term and versatile utility.

## Supplementary Data

Video 1 Illustration of technical steps of currently reported extra-articular soft arthroscopic Latarjet technique (ex-SALT) for management of anterior gleno-humeral (GH) instability in the right shoulder while arthroscopically viewing from the standard posterior GH portal and seating the patient in beach-chair position. Following arthroscopic diagnosis of soft tissue lesions of anterior GH instability and exclusion of engaging Hill-Sachs lesion and critical anterior glenoid bone loss, ex-SALT is performed according to the following technical steps: 1. Through a 5-cm vertical skin incision centered at crossing point of lower border of pectoralis major (PM) and antero-medial border of the arm, subcutaneous tissue is dissected to expose the fascia (covering PM), which is then swept off by a gauze sponge to help visualize the lower border of PM. 2. Antero-medial side of the upper arm is explored by blunt dissection until identification of the long head of biceps (LHB) tendon, which can be readily rolled by surgeon’s fingertip against the proximal humerus. Then, LHB tendon is stitched at the lower border of PM by an absorbable suture. The latter is used as a stay suture (i.e., prevents uncontrolled escape of LHB tendon into the arm following tenotomy) and as a marking suture for future reestablishment of biceps muscle length-tension relationship. 3. Under arthroscopic visualization from the posterior GH portal and through the anterior mid-glenoid portal, LHB tendon is tenotomized from its superior labral origin by combination of the soft tissue biter and motorized shaver blade. 4. Free end of LHB tendon is pulled out (aided by the stay suture) from under the cover of PM, trimmed off and whip-stitched by a couple of # 2 nonabsorbable sutures of different colors over a length of 1 cm for each suture with an intervening whip-stitch-free distance of 1 cm. Whip-stitching should be performed in a way that free ends of the sutures are kept 3 cm apart. 5. Anterior mid-glenoid cannula is removed to provide a room for a blunt arthroscopic trocar/sheath to be passed deep to lower border of PM to appear through the anterior mid-glenoid portal in order to deliver whip-stitched LHB tendon (using shuttle sutures passed through the arthroscopic sheath) out of anterior mid-glenoid portal. 6. At a distance of 1 cm from its upper border and of about 2 cm from its insertion into the lesser tuberosity, SSC tendon is pierced (under arthroscopic visualization) by a sharp, long straight artery clamp (or alternatively by a sharp arthroscopic trocar); creating a SSC hole that can be widened by motorized shaver blade into a SSC window/split of about 1-1.5 cm in length. 7. An arthroscopic ring forceps is used to pass whip-stitching sutures through the anterior mid-glenoid cannula (from its articular to diaphragm ends), which, in turn, is placed back into its corresponding portal. Using an arthroscopic ring forceps (or suture grasper), free ends of the proximal whip-stitching sutures are passed through SSC window to be delivered behind SSC. An arthroscopic ring forceps is then used to retrieve the delivered sutures above SSC tendon and then out of the anterior mid-glenoid cannula. Retrieved sutures are pulled until whip-stitched LHB tendon is shuttled through SSC window into the GH joint. 8. A limb of retrieved proximal whip-stitching sutures is tied to a limb of the distal whip-stitching sutures and the knot is pushed down the cannula by a knot pusher until it rests on LHB tendon. This knot is further secured by 6 alternating half hitches. The remaining untied sutures are then tied in a similar fashion. Knot tying eventually results in suturing whip-stitched end of LHB tendon over itself around a 1-cm cuff of upper border of SSC tendon. During knot tying, the marking nonabsorbable suture should be kept level with lower border of PM for accurate reestablishment of biceps muscle length-tension relationship. This tenodesis can be further secured by additional simple stitches (using a direct suture passer) to firmly hold LHB and SSC tendons together. 9. Under arthroscopic visualization, integrity of soft tissue tenodesis of LHB to SSC tendon is checked by probing, axial loading of LHB tendon by an artery clamp, and 90°-90° provocative GH positioning; the latter 2 maneuvers are helpful in exhibiting the hammock mechanism of SSC in restoration of GH stability. 10. Following ex-SALT; concurrent GH restabilization procedures (e.g., standard Bankart repair and remplissage capsulo-tenodesis) can be performed.

ICMJE author disclosure forms
